# Developmental origins of immunometabolic health

**DOI:** 10.1097/IN9.0000000000000075

**Published:** 2026-01-30

**Authors:** Yem J. Alharithi, Gladys Smith, Alina Maloyan

**Affiliations:** 1 Knight Cardiovascular Institute, Oregon Health & Science University, Portland, OR, USA; 2 University of Pennsylvania, Philadelphia, PA, USA

**Keywords:** pregnancy, immunometabolism, DOHAD, maternal obesity, developmental programming, inflammation, dipeptidyl peptidase 4

## Abstract

Obesity during pregnancy not only affects maternal health but also puts offspring at high risk of obesity and of metabolic and cardiovascular diseases later in life, often regardless of their own lifestyle choices. This process, known as developmental programming, is increasingly being recognized as a critical factor shaping long-term health outcomes. In addition, a growing body of research highlights the importance of immunometabolism, the metabolism of immune cells, in the pathogenesis of obesity, metabolic disorders, cardiovascular disease, and cancer. Although the effects of maternal obesity on offspring health have been well documented in both epidemiological studies and preclinical models, its influence on the developing immune system and immunometabolic pathways is only beginning to be revealed. In this review, we explore the metabolic and inflammatory dimensions of developmental programming and discuss how an adverse maternal environment may shape the offspring’s immunometabolic landscape.

## Graphical abstract

**Figure:**
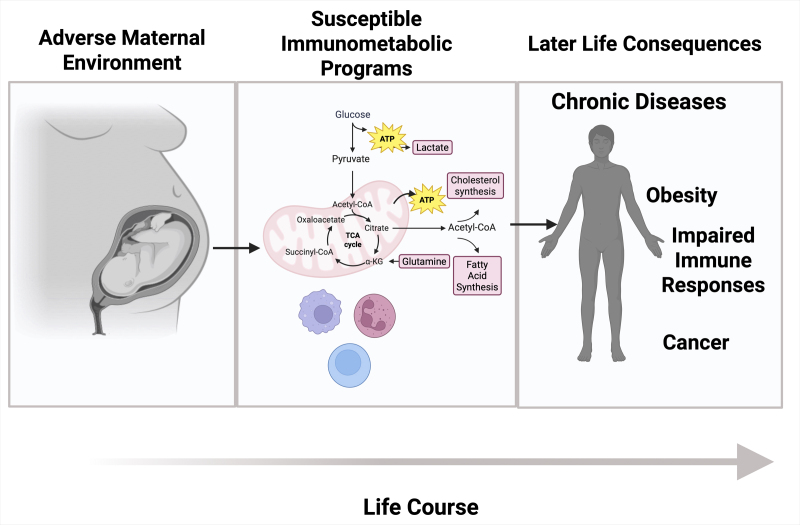

## 1. Introduction

In 1997, the World Health Organization (WHO) officially recognized obesity as a global epidemic for the first time, marking a pivotal shift in public health priorities—from traditional concerns such as undernutrition and infectious diseases to the rising burden of excess weight ^[1]^. More than a quarter-century later, obesity remains a major public health challenge in both developed and developing countries. According to a recent WHO report, 13% of the global adult population, more than 1.9 billion people, is now classified as obese, nearly triple the percentage reported in 1975 ^[1]^. Crucially, overweight and obesity are major risk factors for a range of chronic diseases, including cardiovascular disease and cancer, which are leading causes of mortality worldwide ^[2]^.

As the percentage of obesity among adults has increased, so too has the percentage of obesity among women of childbearing age, particularly in high-income and middle-income countries ^[3]^. In the United States, three out of ten women are obese prior to becoming pregnant; specifically, the percentage has risen from an already-alarming 26.1% of pregnant women in 2016 to 29% in 2019, a trend mirroring the previous decade in the United Kingdom ^[4–6]^. This is a concerning trend, as obesity has recently been shown to have long-term adverse health consequences not just for the pregnant women, but for their children as well ^[7]^.

It is now well established that exposure to maternal obesity increases the risk of metabolic disease in adult offspring, a phenomenon known as developmental programming ^[8,9]^. Substantial epidemiological evidence supports this association ^[9]^. For example, higher maternal pre-pregnancy body mass index (BMI) has been linked to increased insulin resistance and hypertension in offspring ^[8,10]^. A meta-analysis of 14 studies identified a significant association between maternal obesity and an increased risk of asthma in offspring ^[11]^. In addition, a cohort study of 2,000 individuals demonstrated a positive correlation between maternal and offspring BMI ^[12]^. Children born to obese mothers are also at increased risk for pediatric infectious morbidity, including respiratory and viral infections ^[13]^.

Considering that maternal obesity impacts both immune and metabolic functions of the offspring, it is not surprising that its effects on immune cell metabolism have emerged as an important focus in recent years. Numerous aspects of immune cell metabolism have been extensively reviewed elsewhere ^[14–17]^ and are not the primary focus of this review. Instead, we summarize here general knowledge related to the developmental programming. The objective of this review is to synthesize current evidence on how maternal environments reprogram immunometabolic function in the offspring. We integrate findings from human pregnancy and neonatal cohorts, cord blood, and placental studies, and animal models to argue that adverse maternal conditions shape immunometabolic development across critical windows of fetal and early postnatal life. The scope encompasses both innate and adaptive immune lineages, key metabolic pathways governing immune cell function, the persistence of immunometabolic alterations into childhood and adulthood, and their contribution to adult cardiometabolic diseases.

## 2. Pandemic of obesity and metabolic diseases

The worldwide epidemic of obesity has led to dramatic increases in the prevalence of associated metabolic diseases, which now represent leading causes of global morbidity and mortality, primarily through their association with cardiovascular diseases. According to American Heart Association, cardiovascular diseases (CVD) were responsible for more than 900,000 deaths in 2022, making CVD the leading cause of death ^[18]^. Evidence suggests that many of the co-morbidities of obesity, including type 2 diabetes, cardiovascular diseases, asthma, and cancer, are related to the generation of low-grade, chronic inflammation ^[19–21]^.

In 2009, three publications established critical roles for both inflammatory and regulatory T cells in mediating and promoting adipose tissue inflammation and altering insulin sensitivity in obesity ^[22–24]^. These reports demonstrated that obesity is associated with an increase in pro-inflammatory CD4^+^ T_H_1 cells in visceral adipose tissue (VAT), an event that precedes the accumulation of macrophages ^[24,25]^. In weight cycling models, CD4^+^ effector memory T cells are likewise increased prior to weight regain ^[26,27]^. Overall, obesity is associated with both systemic inflammation and an influx of pro-inflammatory immune cells into the VAT and liver ^[28–30]^.

Adipose tissue inflammation has a unique role in the pathology of obesity. Where a bacterial or viral infection provokes a sharp, acute rise in activated immune cells and pro-inflammatory cytokine levels in the blood and affected tissue, the immune response in obesity is characterized by a lower-magnitude but more sustained activation of immune cells and production of pro-inflammatory cytokines in diverse tissue types throughout the body ^[31]^. The seminal work in this field was performed in 1993 by Hotamisligil and colleagues, who showed that obesity increased tumor necrosis factor-α (TNF-α) in the adipose tissue of both mice and humans compared with lean controls ^[32,33]^. In searching for a mechanistic explanation for this discovery, a wealth of clinical and animal models were generated revealing that obesity, characterized by an increase in abdominal adiposity, free fatty acids, and apoptotic adipocytes, is associated with a significant increase in bone-marrow-derived macrophages, T cells, and B cells; a phenotypic switch of tissue-resident macrophages from the broadly anti-inflammatory state to the pro-inflammatory state; and a reduction in anti-inflammatory regulatory T cells ^[24,34–36]^. Consequently, obesity is also associated with higher serum and adipose tissue levels of interleukins (IL-6 and IL-1β), TNF-α, monocyte chemoattractant protein-1 (MCP-1), and other pro-inflammatory cytokines, mediators, and chemokines ^[36–41]^. Notably, whereas inflammation in the liver is resolved upon weight loss, adipose tissue seems to have an obesogenic memory and retains its inflammatory state despite weight loss ^[30]^. Several potential mechanisms have been proposed to explain inflammation in obesity: (1) Changes in microbiota ^[42–44]^; (2) Increase in lipid species or free fatty acids due to diet or obesity ^[45–48]^; and (3) Triggering of an inflammatory response by adipose tissue expansion, via either adipocyte death ^[49–51]^, adipose tissue hypoxia ^[52–54]^, or interaction between adipocytes and the extracellular matrix ^[55,56]^. Importantly, subsequent investigations into the mechanisms of metabolic disorders have implicated increased expression of pro-inflammatory cytokines as a causative factor in the development of altered metabolic states; in particular, Lee et al ^[57]^ showed that inflammation induced by a high-fat diet is necessary for the maintenance of long-term insulin resistance, and insulin resistance has in turn been demonstrated a significant risk factor for cardiovascular diseases ^[58,59]^.

## 3. Developmental programming of cardiometabolic disease

Epidemiological studies provide strong evidence linking maternal obesity with increased offspring risk of coronary heart disease, stroke ^[60]^, diabetes ^[61]^, asthma ^[11]^, and cancer ^[62–64]^. Maternal obesity programs offspring toward increased adiposity and metabolic dysfunction ^[65,66]^, thereby perpetuating a transgenerational cycle of obesity and its associated health consequences ^[67]^. A large record-linkage cohort study of 37,709 individuals in Scotland (ages 34–61) demonstrated a significant association between maternal overweight or obesity and heightened cardiovascular events in offspring, accompanied by increased all-cause mortality ^[68]^. Similarly, analysis of 13,345 men and women in the Helsinki Birth Cohort Study revealed that higher maternal BMI was positively associated with offspring all-cause mortality, cancer incidence and mortality, cardiovascular morbidity and mortality, and diabetes, independent of childhood and adult socioeconomic status ^[12]^. Collectively, findings from these and numerous other cohort studies over the past several decades strongly support a mechanistic link between adverse maternal environments and cardiometabolic disease risk in offspring ^[34,69]^. These observations have fueled growing interest in clinical and experimental animal studies investigating what is now widely recognized as the “Developmental Origins of Health and Disease” (DOHaD) ^[70]^.

## 4. Maternal environment and offspring’s immunity in health and disease

In a normal human pregnancy, inflammatory signaling pathways are activated in the first and third trimesters, as inflammation is essential for successful implantation and development of the fertilized egg/embryo in the uterine/endometrial epithelium ^[71,72]^. In mice, most adult tissue-resident macrophages, such as Kupffer cells in the liver, microglia in the brain, Langerhans cells in the epidermis, and alveolar macrophages in the lung, do not arise from adult hematopoietic stem cells (HSCs), but instead originate during embryonic development. These cells derive from erythro-myeloid progenitors (EMPs), a distinct myeloid lineage generated through a Tie2^+^ (Tek) and Csf1r^+^ developmental pathway. EMPs first emerge in the yolk sac around embryonic day (E) 8.5 and subsequently migrate to the nascent fetal liver by E10.5. Within the fetal liver, EMPs give rise to multiple myeloid lineages, including macrophages, monocytes, granulocytes, and fetal erythrocytes, until at least E16.5 ^[73,74]^. Developmental fate-mapping studies have revealed that hematopoiesis in the fetal liver occurs in sequential waves, beginning with yolk sac derived EMPs and later transitioning to HSC-derived hematopoiesis ^[73,74]^. Importantly, these studies established yolk sac EMPs as a major and common developmental source of tissue-resident macrophages across organs. More recent barcode-based lineage-tracing approaches have further refined this model by demonstrating that tissue-resident macrophages establish residency very early during development and are maintained throughout life largely through local self-renewal ^[75]^. These macrophage populations exhibit minimal migration between organs, and even limited redistribution within individual tissues ^[75]^. The remarkable consistency of these lineage patterns across animals underscores the existence of intrinsic, tightly regulated developmental programs that govern tissue-specific macrophage identity and maintenance.

Resident immune cells actively regulate angiogenesis, branching morphogenesis, neuronal circuit refinement, epithelial barrier formation, and programmed cell death through the secretion of growth factors, cytokines, and morphogens such as VEGF, TGF-β, and Wnt ligands ^[76–78]^. Importantly, tissue immune cells also shape local metabolic environments and mitochondrial maturation, thereby linking immune colonization directly to long-term tissue metabolic function ^[79]^. Using peripheral blood from pregnant women at term, Rees et al demonstrated that monocytes lose anti-inflammatory properties and acquire a pro-inflammatory phenotype, accompanied by reductions in monocytes mitochondrial mass and respiration ^[80]^. Perturbations in the intrauterine environment, including maternal obesity, inflammation, or hypoxia, disrupt immune cell seeding and imprint persistent epigenetic and immunometabolic changes in tissue-resident immune populations, programming susceptibility to cardiometabolic, inflammatory, and neurodevelopmental diseases across the lifespan ^[79,81,82]^.

Maternal malnutrition and stress during pregnancy increase expression of inflammatory markers beyond normal levels. By a multi-omics approach, Musillo et al demonstrated fetal sex–specific adaptations to prenatal stress and maternal high-fat diet that distinctly targeted the placenta and fetal brain ^[83]^. Placental function was selectively disrupted in male fetuses, with the emergence of signaling pathways associated with cardiometabolic risk ^[83]^. In contrast, the fetal brain was predominantly affected in females, exhibiting increased expression of inflammation-related gene programs ^[83]^. Ramsay et al ^[84]^ reported elevated plasma levels of IL-6 and sensitive C-reactive protein during the first trimester in obese vs. normal weight pregnant women, and Aye et al ^[85]^ revealed increased circulating TNF-α in maternal serum to correlate with increased BMI. Hayes et al recently demonstrated that maternal immune activation in mice induces a long-lasting reduction in microglial immune reactivity in the offspring across the developmental trajectory ^[86]^. This attenuated response was associated with changes in chromatin accessibility and decreased transcription factor occupancy at open chromatin sites ^[86]^.

Exposure to chronic, low-grade inflammation induces a persistent alteration in metabolic state with both immediate and long-term consequences for the health of the obese mother and her child. For the mother and fetus, obesity during pregnancy is associated with increased risk of developing gestational diabetes melitus (GDM) and pre-eclampsia ^[87,88]^. Meta-analyses by Chu et al and Santos et al revealed a strong BMI-dependent increase in GDM risk, with odds ratios of approximately 2, 4, and 8 in overweight, obese, and severely obese women, respectively, compared with normal-weight controls ^[89,90]^. The adjusted risk of developing preeclampsia was shown to be doubled for overweight mothers and almost tripled for obese mothers ^[91]^. While the underlying biological processes that drive these adverse effects are poorly understood, it is likely that the altered maternal metabolic and inflammatory dysregulations play a role.

Persistent elevations in circulating glucose, saturated lipids, and lipoproteins drive chronic, low-grade inflammation in obese mothers ^[92,93]^. This inflammatory and hyperglycemic intrauterine environment profoundly reshapes placental structure, immune complexity, and key signaling pathways at the feto-maternal interface ^[94–98]^. Aberrations in placental immune cell profile (macrophage, regulatory T cell, and natural killer cell abundance and phenotype), in turn, could disrupt the angiogenesis and vascular remodeling that is pivotal for the development of a healthy placenta-uterine interface ^[99,100]^.

For the offspring, both clinical and animal model studies have demonstrated pre-gravid obesity to modulate immunity and infectious-disease-related outcomes. While clinical studies are naturally limited in their investigative scope, several have profiled cord blood, which is established to be an excellent representation of the fetal immune system at birth ^[101]^, and have found changes in immune cell frequency and phenotype ^[92,102–104]^. In particular, a 2015 study by Wilson et al ^[92]^ found babies born to obese mothers to have fewer eosinophils and CD4^+^ T helper cells compared to babies born to lean mothers, as well as increased plasma levels of interferon α2 and IL-6. Gonzalez-Espinosa et al ^[103]^ likewise found CD3^+^, natural killer, and CD8^+^ regulatory T cells to be elevated in babies born to obese mothers. Animal model studies have yielded similar results, with Zhu et al and Yan et al ^[104,105]^ demonstrating in sheep that maternal obesity induces inflammation and enhances expression of proinflammatory cytokines, Toll-like receptors (TLRs) 2 and 4, and the inflammatory NF-кB and JNK signaling pathways in the large intestine of fetal and offspring ewes suggesting a potential cause-and-effect relationship between maternal obesity and development of metabolic syndrome in the offspring of obese mothers.

From a functional standpoint, maternal obesity has also been linked to altered offspring immune responses. Wilson et al ^[92]^ showed that myeloid dendritic cells and monocytes isolated from the umbilical cord blood of obese and overweight mothers produce less TNF-α and IL-6 in response to TLR1 and TLR2 and TLR4 agonists compared to cells from lean mothers. Sureshchandra et al ^[106]^ recently extended this line of work, demonstrating that umbilical cord blood monocytes from obese mothers have dampened inflammatory responses to bacterial and viral pathogens. These findings are in line with reports of poor response to infection and increased neonatal intensive care unit visits in the neonates of obese mothers and highlight the damaging effect of maternal obesity on offspring immunity ^[107,108]^. Emerging research is increasingly focused on the bidirectional crosstalk between the maternal metabolic milieu and the developing immune system of the offspring, with particular emphasis on the offspring’s immunometabolic reprogramming as result of maternal obesity.

## 5. Immunometabolism and developmental programming

Much remains to be elucidated regarding the relationships between maternal environment and the offspring’s immunometabolism; for example, do fetal immune cells respond to maternal obesity by committing to distinct metabolic pathways? And if so, what are the long-term consequences of these metabolic choices? This far, only a few studies have established a direct link between maternal environment and immunometabolic changes in offspring. Using a mouse model, Huang et al ^[109]^ recently reported that offspring exposed to maternal obesity develop fatty liver disease driven by developmental programming of Kupffer cells (KCs), liver-resident macrophages that colonize the organ early during embryogenesis. Specifically, the metabolically reprogrammed KCs in neonates born to obese mothers secrete apolipoproteins that promote lipid uptake by hepatocytes; depletion of these KCs, followed by replenishment with naïve monocytes, rescued the fatty liver phenotype ^[109]^. The authors further interrogated the underlying immunometabolic mechanisms, showing that genetic ablation of hypoxia-inducible factor-1α (HIF-1α) in macrophages during gestation prevented the metabolic reprogramming of KCs from oxidative phosphorylation to glycolysis, thereby protecting against fatty liver disease in the offspring ^[109]^. Other studies demonstrate that maternal high-fructose consumption increases GLUT5-dependent fructose uptake and catabolism in offspring microglia, leading to accumulation of fructose-6-phosphate, reduced microglial phagocytosis, impaired clearance of dying cells in the developing prefrontal cortex, and ultimately persistent cognitive deficits ^[110]^. Data from other groups further suggest that maternal HFD elevates fetal exposure to endotoxin (lipopolysaccharide, LPS), which primes microglial TLR4 signaling and shifts microglial functional states ^[111]^. Notably, these effects are sex-specific: male offspring exhibit excessive microglial pruning of serotonergic neurons, whereas females are largely spared ^[111]^.

In a recent report from our team, Alharithi et al utilized a mouse model of maternal high-fat diet–induced obesity to demonstrate significant metabolic, lipidomic, and transcriptomic perturbations in bone marrow myeloid cells of newly weaned offspring of obese mothers ^[112]^. A particularly surprising finding was that, despite increased adiposity in both mothers and offspring, a marked reduction in triglyceride levels was observed only in offspring bone marrow myeloid cells ^[112]^. This reduction was accompanied by increased mitochondrial respiration, suggesting enhanced triglyceride breakdown and mobilization through lipolysis. Supporting this notion, the gene *lipoprotein lipase* was significantly upregulated in offspring bone marrow myeloid cells ^[112]^. All these findings point to early-life reprogramming of immune cell metabolism by maternal obesity and raise important questions about the long-term consequences for immune function and disease susceptibility in later life.

Nutrition plays a central role in immunometabolism, and immune cell metabolism is particularly sensitive to nutritional dysregulation, including both undernutrition and overnutrition ^[113]^. Among the key mediators of this crosstalk are adipokines, bioactive molecules secreted by adipose tissue that facilitate communication between adipose depots and peripheral organs, including the immune system ^[114]^. For example, leptin, which is elevated in pregnancies complicated by maternal obesity ^[115]^, exerts pleiotropic effects on the immune system. Most immune cells express the leptin receptor, and leptin signaling has been demonstrated to be critical for hematopoietic cell development, immune cell proliferation and survival, and pro-inflammatory function ^[116]^. Another adipokine, adiponectin, has insulin-sensitizing effects but circulates at lower levels in women who are obese or have gestational diabetes ^[117]^. Functionally, adiponectin suppresses pro-inflammatory (LPS/IFN-γ–induced) macrophage activation and the production of pro-inflammatory cytokines such as TNF-α, MCP-1, and IL-6, while simultaneously promoting alternatively activated (IL-4/IL-13–induced) macrophage proliferation and the expression of anti-inflammatory markers including arginase-1, macrophage galactose-type lectin-1, and IL-10. These actions underpin adiponectin’s well-recognized anti-inflammatory properties ^[118]^.

Still another key adipokine, dipeptidyl peptidase 4 (DPP4), is produced by hepatocytes, adipocytes, intestinal K cells, placental cytotrophoblasts, immune cells, and endothelial cells, among others. It exists in two isoforms, soluble (s-DPP4) and membrane-bound (m-DPP4) ^[119]^, which serve distinct roles: m-DPP4 functions as a receptor that regulates intercellular communication, while s-DPP4 mediates intercellular and inter-organ crosstalk by cleaving protein substrates in bodily fluids such as plasma and/or by binding to receptors ^[120]^. DPP4 was initially implicated in regulation of the biological activity of hormones and chemokines such as glucagon-like peptide-1 and glucose-dependent insulinotropic polypeptide; however, accumulating evidence suggests that it is also involved in numerous other processes, including immune responses, lipid metabolism, and mitochondrial function ^[120–124]^. In obesity, DPP4 has been implicated in promoting fat retention and metabolic dysfunction ^[125]^. Epidemiological studies demonstrate that plasma DPP4 activity is elevated in obese children ^[126]^, obese adults ^[127]^, and patients with type 2 diabetes ^[127–130]^, hypertension ^[131]^, and renal disease ^[132]^. Importantly, elevated plasma DPP4 activity in childhood has emerged as a potential early biomarker of increased risk for later-life obesity ^[127,130,133]^. Supporting the concept that DPP4 actively contributes to obesity progression, clinical studies show that DPP4 inhibitors improve weight regulation ^[134–140]^, systemic metabolism ^[141–146]^, and inflammatory responses ^[147–154]^, processes that are progressively dysregulated in obesity.

In the context of maternal obesity, our team has reported ^[155]^ that plasma DPP4 activity is elevated in obese pregnant women and in their male, but not female, offspring, indicating a sex-dependent response to maternal obesity. Specifically, using a mouse model of high-fat diet–induced maternal obesity, we showed that treatment of HFD-fed mothers with the DPP4 inhibitor sitagliptin during third trimester and lactation delayed the onset of obesity and metabolic disorders in male offspring, whereas female offspring were unaffected ^[155]^. While the effects of DPP4 inhibition on immune cell metabolism remain to be fully elucidated, both clinical and experimental studies point to DPP4 inhibitors having immunomodulatory roles. For instance, Nishida et al demonstrated in *Apoe−/−* mice, a model of human atherosclerosis, that the DPP4 inhibitor linagliptin enhances anti-inflammatory macrophage polarization in the bone marrow and aorta without affecting pro-inflammatory macrophages ^[156]^. Similarly, in LDL receptor knockout mice fed a high-fat diet, another model of atherosclerosis, Shah et al reported the DPP4 inhibitor alogliptin to reduce macrophage infiltration in the VAT, suppress expression of pro-inflammatory genes, decrease aortic plaque size, and markedly reduce intraplaque macrophage content ^[157]^. Clinical data also support immunomodulatory effects of DPP4 inhibitors. In diabetic patients, sitagliptin treatment is associated with a higher proportion of T_H_2 cells and a lower proportion of T_H_17 cells compared with controls ^[158]^. Aso et al further showed 12 weeks of sitagliptin in type 2 diabetic patients to reduce both the number and percentage of circulating T_H_17 and Treg cells, though the T_H_17/Treg ratio remained unchanged ^[159]^.

These findings raise the intriguing possibility that the shifts in immune cell subpopulations observed with DPP4 inhibition may be driven by underlying alterations in glycolytic, mitochondrial, and lipid metabolic pathways. Exploring these mechanisms in the context of maternal obesity could provide critical insights into how DPP4 inhibition shapes immune–metabolic interactions.

## 6. Concluding remarks and future directions

Accumulating evidence suggests that immunometabolism links early-life environmental exposures to lifelong disease risk, revealing how immune cell energy metabolism and substrate utilization can be reprogrammed during development. Adverse in utero and early postnatal conditions, including maternal obesity, gestational diabetes, hypoxia, and chronic inflammation, rewire immune metabolic pathways potentially through stable epigenetic and energetic adaptations, giving rise to persistent inflammatory bias and altered immune responsiveness that promote cardiometabolic diseases. Despite major advances, critical gaps remain in defining the cell-type–specific metabolic checkpoints, temporal windows of vulnerability, and sex-specific mechanisms governing immune programming across tissues. Future directions will integrate single-cell and spatial multi-omics phenotyping with developmentally timed experimental models and longitudinal human cohorts to precisely map how early immunometabolic perturbations translate into adult disease. Identifying reversible versus irreversible programming nodes will be essential for developing prevention strategies and targeted immunometabolic therapies to interrupt the programming of cardiometabolic diseases.

Immune cells adapt to adverse environments such as inflammation, hypoxia, nutrient excess or deprivation, and cancer by reprogramming their metabolism and gene expression, a plasticity that enables them to maintain function, ensure survival, and coordinate appropriate responses under stress. Fetuses do the same, adjusting their immunometabolic profile to withstand the challenges of an adverse maternal environment. What remains unclear is whether these fetal adaptations are causal drivers of later outcomes or merely consequences of the broader “programming” process. That is, while immunometabolic adjustments may support fetal survival in a highly inflammatory and insulin-resistant intrauterine environment, they may also predispose offspring to impaired immune defense and increased susceptibility to chronic diseases later in life. If this is the case, when and where does the maternal environment cross the threshold from adaptive to detrimental? And what metabolic pathways can be targeted to restore normal immune function in the offspring of obese mothers?

## Author contributions

YJA and GS performed the literature search; YJA and AM wrote the review; AM supervised data gathering and integration and headed the writing.

## Conflict of interest

The authors declare no competing interests in this manuscript.

## Acknowledgements

AM work is supported by National Institute of Health grants NIH R01HL170097, R01HL164474, and R21HD118370.
